# Role of the prion protein family in the gonads

**DOI:** 10.3389/fcell.2014.00056

**Published:** 2014-10-02

**Authors:** Aurélie Allais-Bonnet, Eric Pailhoux

**Affiliations:** Institut National de la Recherche Agronomique, UMR 1198, Biologie du Développement et ReproductionJouy-en-Josas, France

**Keywords:** prion, Doppel, Shadoo, PRT, reproduction, gonads

## Abstract

The prion-gene family comprises four members named *PRNP* (PRP^c^), *PRND* (Doppel), *PRNT* (PRT), and *SPRN* (Shadoo). According to species, *PRND* is located 16–52 kb downstream from the *PRNP* locus, whereas *SPRN* is located on another chromosome. The fourth prion-family gene, *PRNT*, belongs to the same genomic cluster as *PRNP* and *PRND* in humans and bovidae. *PRNT* and *PRND* possibly resulted from a duplication event of *PRND* and *PRNP*, respectively, that occurred early during eutherian species divergence. Although most of the studies concerning the prion-family has been done on PRP^c^ and its involvement in transmissible neurodegenerative disorders, different works report some potential roles of these proteins in the reproductive function of both sexes. Among them, a clear role of *PRND*, that encodes for the Doppel protein, in male fertility has been demonstrated through gene targeting studies in mice. In other species, Doppel seems to play a role in testis and ovary development but its cellular localization is variable according to the gonadal developmental stage and to the mammalian species considered. For the other three genes, their roles in reproductive function appear ill-defined and/or controversial. The present review aimed to synthesize all the available data on these prion-family members and their relations with reproductive processes, mainly in the gonad of both sexes.

## Introduction

**Figure 1:**
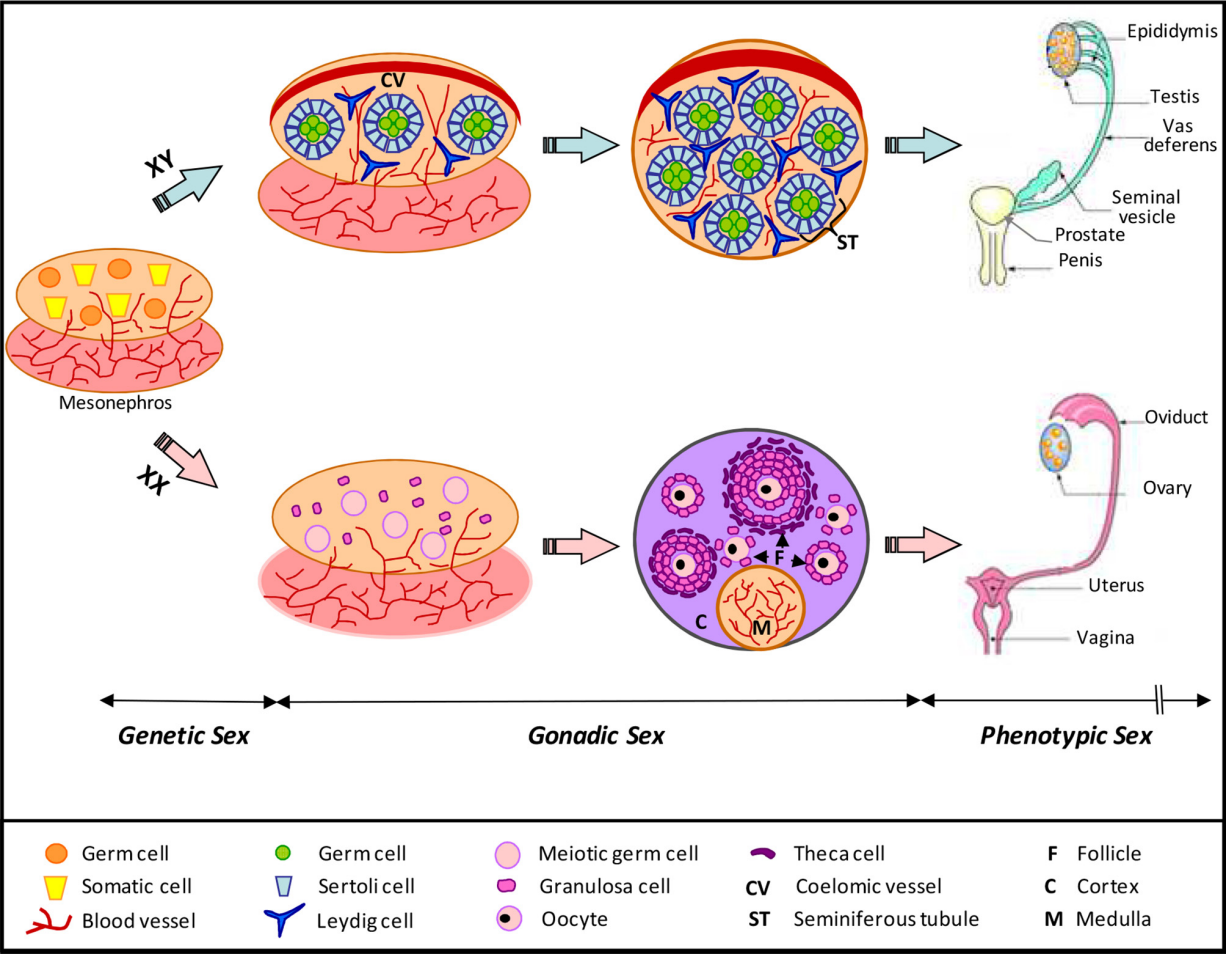
**Schematic representation of mammalian gonad differentiation**. The genetic sex directs the development of the bipotential gonad toward a male or female gonadal fate. Three cellular types are present in differentiating gonad: somatic supporting cells, somatic steroid-producing cells, and germinal cells. Male and female somatic supporting cells have a common origin and differentiate respectively in Sertoli and granulosa cells. Steroidogenic cells will differentiate in Leydig cells in testis and theca cells in ovary. Testicular-specific features include the formation of the coelomic arterial vessel and of the seminiferous tubules formed by germ and Sertoli cells. Ovarian-specific features are in a chronological order: (i) entry of germ cells into meiosis, (ii) establishment of cortical and medullar compartments, and (iii) formation of follicles, which contain oocytes surrounded by granulosa cells. In agreement with the genetic sex, the development of the phenotypic sex is achieved with the differentiation of the genital tract: epididymis, vas deferens, seminal vesicle, prostate, and penis in males; oviduct, uterus and vagina in female (Adapted from DeFalco and Capel, 2009; for review).

In Eutherian mammals, the reproductive system is composed by gonads (testes and ovaries) and the genital tract (male: penis, prostate, seminal vesicle, vas deferens, epididymis; female: vulva, vagina, uterus, and oviduct). Gonads produce sex hormones and gametes (sperms and oocytes), whereas the genital tract provides a suitable environment for the maturation and transport of gametes, the fertilization and implantation of the eggs. The differentiation of reproductive organs follows a specific and variable chronology according to species. In every case, the sex determination occurs immediately at fertilization with the addition of male and female gamete genomes. This step determines the genetic sex of the embryo and induces latter on the differentiation of gonads (arising from mesonephros, a transient embryonic kidney) toward a testicular (XY) or an ovarian (XX) differentiating pathway (DeFalco and Capel, 2009; for review; Figure 1). The undifferentiated gonad is composed by a germinal and two somatic cell-lineages. Each somatic line presents a double potentiality and will be turned toward a specific gonadal fate depending of the genes involved in sex determination, with *SRY* (Sex-determining Region of Y) being at the top of them (Kashimada and Koopman, 2010; for review). In somatic cell populations, we distinguish: (1) the supporting cells, which will differentiate into Sertoli cells in males and into follicular (also called granulosa) cells in females (these cells are responsible for the growth and the maturation of the germ line); (2) the steroidogenic cells, which will differentiate in Leydig cells in male and theca cells in female (Figure 1). The phenotypic sex, which depends on the gonadal sex and its hormonal production, is set up in many successive steps during development from early gonad differentiation until adulthood. Testes produce androgens and AMH (Anti-Müllerian Hormone) which are responsible for the differentiation of the genital tract toward the male pathway. Without these hormones the genital tract will differentiate into the female pathway.

Most of the major genes involved in gonad differentiation have been discovered through human genetic studies of DSD (Disorders of Sex Differentiation) cases, but many other genes were found to be expressed in gonads following high throughput mRNA sequencing or other expressional studies. Most of the genes of this last category could putatively be involved in gonadal processes but their role remains to be defined. Among these genes are those of the prion-family. Notably, *PRND* has been shown to be essential for testicular function in several species (Behrens et al., 2002; Paisley et al., 2004; Kocer et al., 2007). The most studied gene in the prion family is *PRNP* which encodes a cell surface glycoprotein, the prion protein (PRP^c^). An infectious isoform of PRP^c^ (PRP^sc^) has been shown to be the major component of “Prion,” the etiological agent of transmissible spongiform encephalopathies (TSEs). These fatal neurodegenerative disorders include Creutzfelt-Jacob disease (CJD) in humans, bovine spongiform encephalopathy and scrapie in bovidae (Prusiner, 1998).

## The prion gene family comprises four members

**Figure 2:**
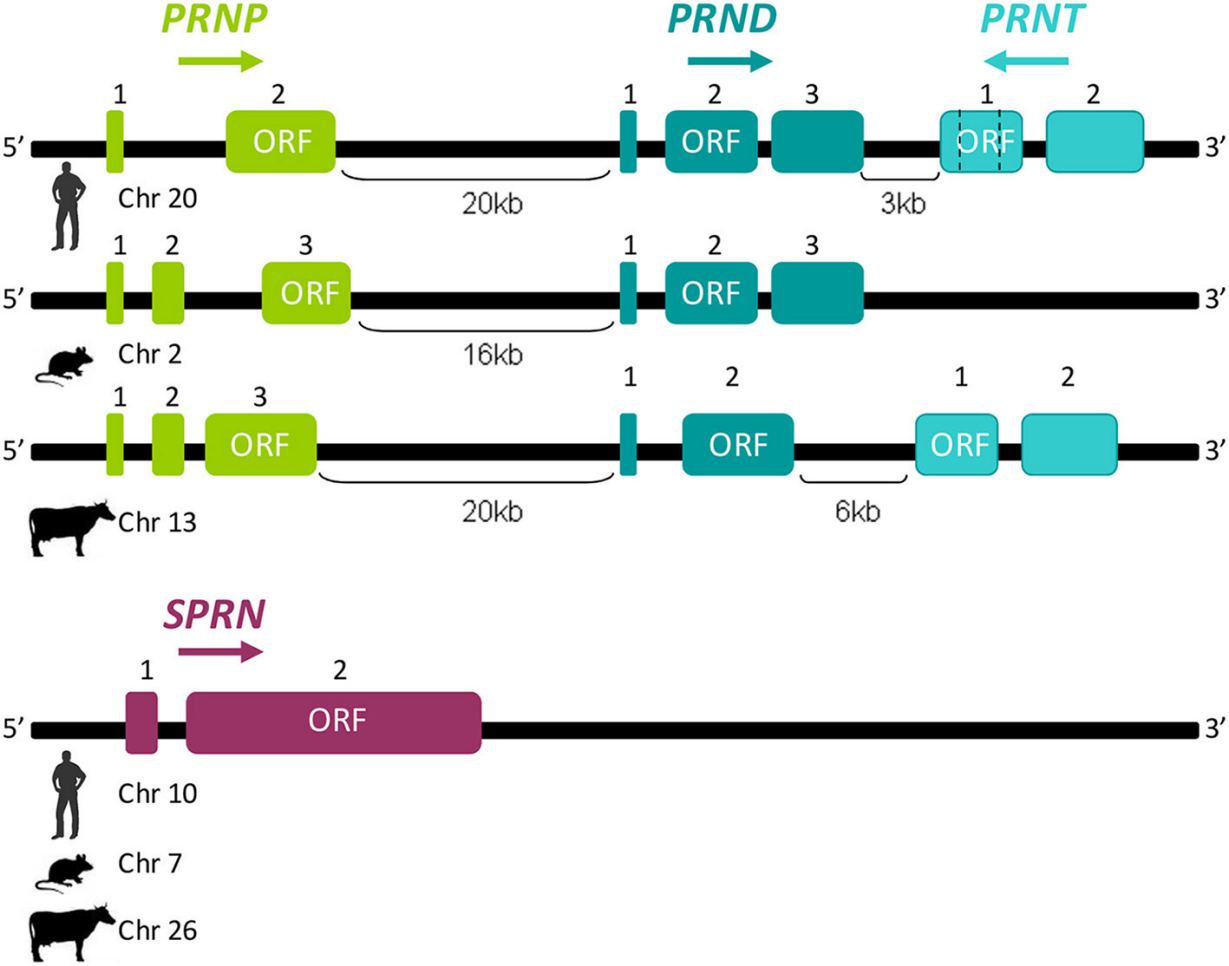
**Schematic structural representation of prion genes family members**. *PRNP*. *PRND*, and *PRNT* are clustered on the same genomic locus. The distance between *PRNP*. *PRND*, and *PRNT* are given in kilobases (kb); according to species, the number of exons could be variable. *SPRN* consist of two exons and is located on another chromosome in all studied species. Arrows indicate the relative orientation of the genes. Chromosomes carrying these genes are annotated near each species symbols. For each gene, the open reading frame (ORF) is indicated on relevant exons.

The “prion gene complex” encompasses four members named *PRNP*. *PRND* (downstream prion protein-like gene), *PRNT* (prion protein testis-specific gene), and *SPRN* (shadow of the prion protein gene). In mouse, sheep, cattle, and rat, the *PRNP* gene is composed of three exons, whereas only two are present in humans (Yoshimoto et al., 1992; Saeki et al., 1996; Lee et al., 1998; Figure 2). Depending on the studied species, *PRND* is located 16–52 kb downstream of *PRNP* and *PRNT* 3 and 6 kb downstream of human and cattle *PRND*, respectively (Moore et al., 1999; Comincini et al., 2001; Essalmani et al., 2002; Makrinou et al., 2002; Kocer et al., 2007). *PRND* and *PRNT* share with *PRNP* the same genomic cluster and possibly result from a duplication, that occurred early during eutherian species divergence, of *PRNP* and *PRND*, respectively. As *PRNP*. *PRND* structure can vary from two to three exons between species, whereas *PRNT* has two exons in humans (Comincini et al., 2001; Makrinou et al., 2002; Figure 2). The same organization of *PRNT* was predicted in cow, sheep, horse, dog and primates whereas this gene seems to be absent in rodents (Premzl et al., 2004; Harrison et al., 2010; Figure 2). *SPRN* is not part of the *PRNP* genomic locus, and is located on another chromosome. *SPRN* comprises two exons and its structure is conserved in fishes and mammals (Premzl et al., 2003; Figure 2). Some of the prion-family genes produce different transcripts of variable compositions and numbers according to the species.

**Figure 3:**
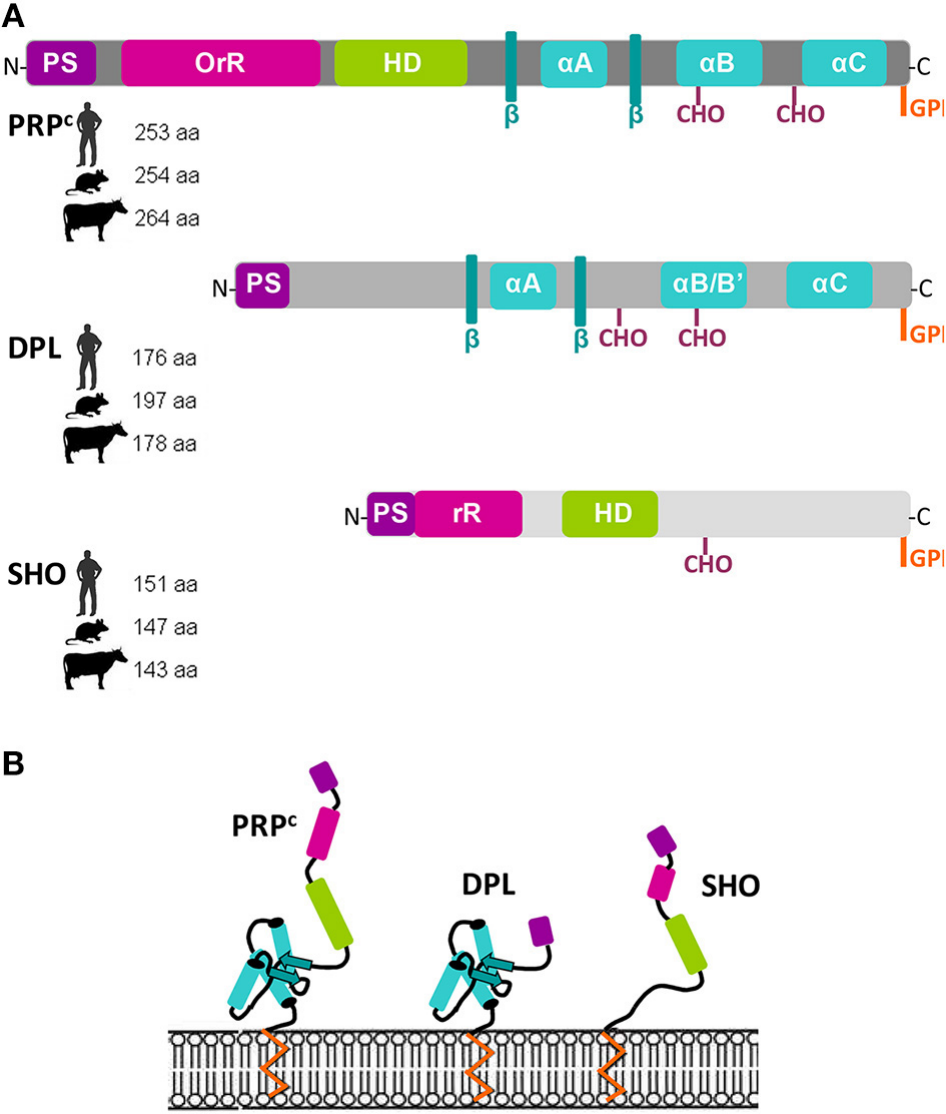
**Predicted structural features of Prion (PRP^c^), Doppel (DPL), and Shadoo (SHO) proteins**. **(A)**: Schematic description of these glycophosphatidylinositol (GPI)-anchored glycoproteins structure includes signal peptide, octarepeat or basic repeat region (OrR or rR), hydrophobic domain (HD), alpha (α)-helices, beta (β)-strands, N-glycosylation sites (CHO). The size of the full-length proteins in amino acids (aa) is annotated near each species symbols. **(B)**: Schematic representation of prion family proteins anchored in a membrane. (Adapted from Schmitt-Ulms et al., 2009; Daude and Westaway, 2011; for review).

The mammalian *PRNP* encodes the PRP protein (PRP^c^) that contains several distinct domains, including an N-terminal signal peptide, an octapeptide repeat domain, a highly conserved hydrophobic segment and a C-terminal hydrophobic region which contains a glycosylphosphatidylinisitol (GPI) anchor (Figure 3A). This glycoprotein possesses two N-linked glycosylation sites and exists in bi-, mono-, and un-glycosylated forms (Figure 3A). Its secondary structure is defined by the presence of three α-helices and two β-strands (Harris, 1999; for review; Figures 3A,B). The mature Doppel protein (DPL) encoded by *PRND* is a protein which resembles a N-terminally truncated PRP^c^ protein lacking the octamer repeats (Figure 3). In contrast, *SPRN* encodes the Shadoo protein (SHO) which shares with PRP^c^ a similar N-terminal region with a basic repeat region and a hydrophobic domain (Watts and Westaway, 2007; for review; Figure 3). Few data are available concerning the structure of PRT, the *PRNT*-encoded protein (Makrinou et al., 2002). No signal peptide was predicted for the 94 aa PRT protein, suggesting it could be an intracellular protein (Premzl and Gamulin, 2007). In bovine, *PRNT* encodes for an N-terminally truncated protein of 55 aa in length, sharing 55% identity with its human counterpart (Kocer et al., 2007).

Although PRP^c^ and SHO are mainly expressed in the central nervous system (CNS), these two proteins were also detected in male and female gonads (Bendheim et al., 1992; Tanji et al., 1995; Young et al., 2011). DPL and PRT are described as testis-specific proteins (Moore et al., 1999; Tranulis et al., 2001; Makrinou et al., 2002; Kocer et al., 2007). Nevertheless, a transient expression of DPL has been observed in brain of neonatal mice, but this protein is absent in the CNS of adult healthy animals (Li et al., 2000).

Many studies have been conducted on the role of DPL in testis differentiation but the implication of the other prion proteins in reproduction still remains subjective.

## Testis-specific prion proteins

**Figure 4:**
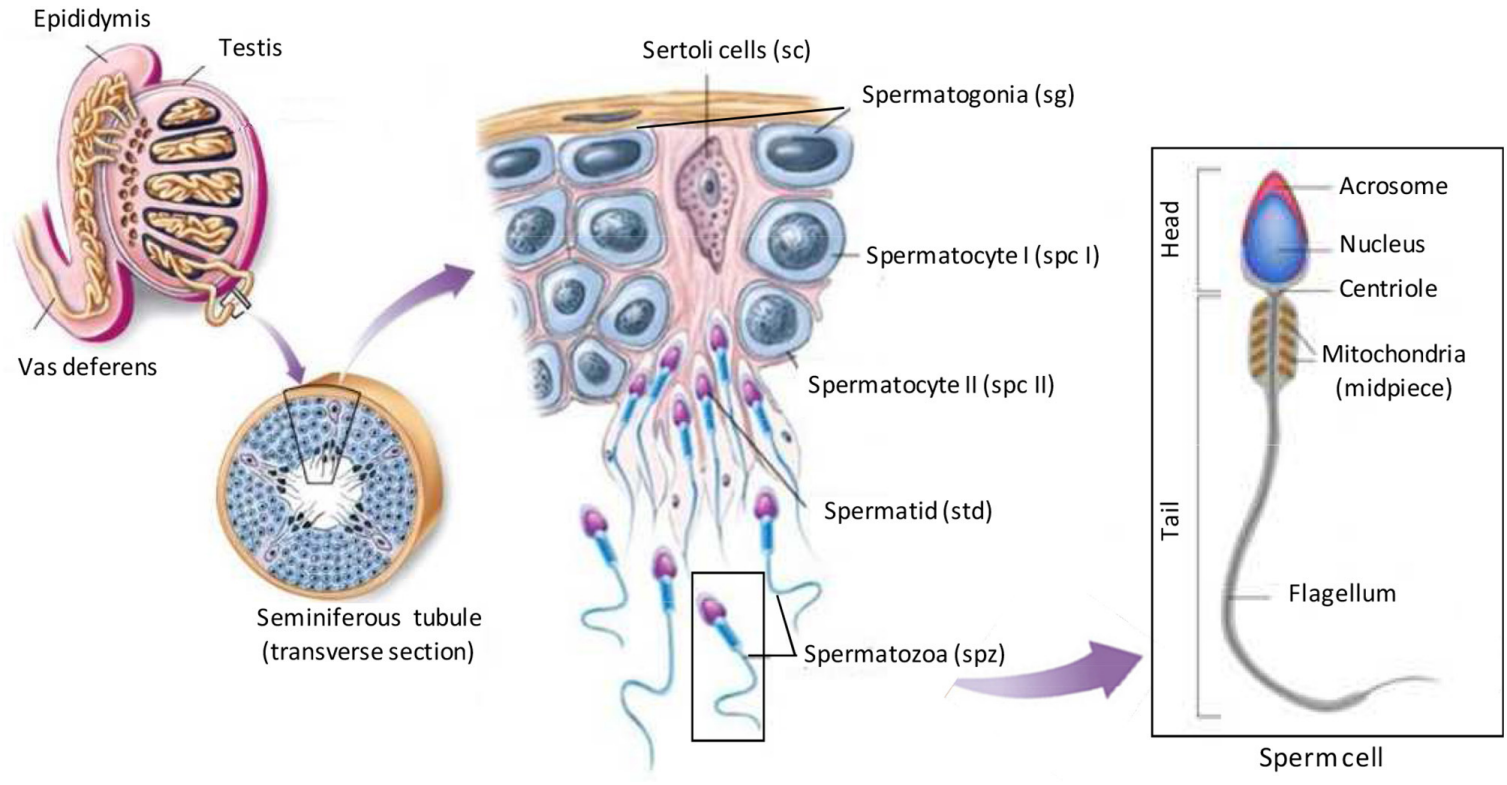
**Illustration of spermatogenesis**. Spermatogenesis occurs within the seminiferous tubules of the testes of a post pubescent male. Diploid primordial germ cells (also called spermatogonia; sg) near the basal lamina of the seminiferous tubules undergo an initial mitotic division to produce diploid primary spermatocytes (spc I). Nearly half the primary spermatocytes produced remain near the basal lamina to continue to divide mitotically, thus allowing spermatogenesis to be continuous during male's reproductive lifespan. Other primary spermatocytes migrate toward the lumen of the seminiferous tubules and begin to undergo meiosis I, resulting in haploid secondary spermatocytes (spc II). These secondary spermatocytes further divide through meiosis II, producing haploid spermatids (std). Mature sperm cells (spermatozoa; spz) capable of fertilizing an egg develop from spermatids through the final stage called spermiogenesis. In this stage specific regions of the spermatid differentiate into the head, mid-piece, and the tail of the sperm cell. Within the head, an acrosomal space is developed which houses specific enzymes required for fertilization. Specialized acrosomal membranes that are pertinent for fertilization also differentiate in the head of the sperm. A flagellum develops as a means of motility near the posterior aspect which is fuelled by the abundance of mitochondria in the mid-piece of the sperm cell. (Adapted from buffonescience9.wikispaces website).

In order to comment on the role of a testis-specific protein, it is necessary to introduce the spermatogenesis process allowing the production of male germ cells, spermatozoa (spz). Three different generations of germ cells are visible at any given time among the epithelial cells of the seminiferous tubule: spermatogonia (sg), spermatocytes (spc), and spermatids (std; Figure 4). During spermatogenesis, developing germ cells undergo several mitotic divisions and two meiotic divisions after translocation through the blood-testis barrier, to the luminal side of the epithelium, that is defined by tight junctions between non-dividing Sertoli cells. The final stage of spermatogenesis, known as spermiogenesis, consists of the complex differentiation of round spermatids into spermatozoa (cell elongation, nucleus condensation, acrosome formation, cytoplasm reduction; Figure 4). Throughout spermatogenesis each cluster of germ cells, derived from a single spermatogonium, is interconnected by cytoplasmic bridges that are important for synchronizing the developing process. Thus, the germ cells can be considered as being isolated cells only after they are released as spermatozoa into the lumen of the seminiferous tubule. Upon release, spermatozoa leave behind excess cytoplasm, in the form of interconnected syncytial chains called cytoplasmic lobes (Sprando and Russell, 1987; Weber and Russell, 1987), which are subsequently engulfed and degraded as residual bodies by the Sertoli cells (Espenes et al., 2006). Then spermatozoa are stored in epididymis and undergo maturation processes necessary to acquire motility and capacity to fertilize. Final maturation is completed in the female reproductive tract where spermatozoa acquire the capacities to fertilize oocytes. This last step called capacitation ended with acrosome reaction which consists on the fusion of the acrosome and oocyte membranes, allowing fertilization.

**Figure 5:**
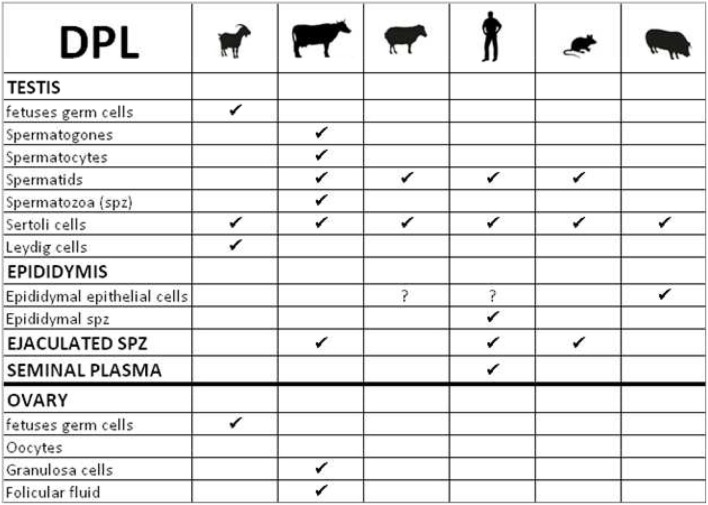
**Table summarizing the cellular localization of Dopple (DPL) in gonads of various species**.

Two members of the prion-gene family could be considered as testis-specific proteins, *PRND* and *PRNT*. *PRND*/DPL expression has been studied mainly during spermatogenesis in many species. The first common observation is that DPL is expressed in Sertoli cells at various concentrations according to the species (Westaway, 2004; Rondena et al., 2005; Serres et al., 2006; Kocer et al., 2007; Figure 5). Nevertheless, in germ cells, the localization of DPL is less comparable between animals. For example in bovidae, DPL is present early in primordial germ cells in goat fetal gonads (Kocer et al., 2007) and in bovine, this protein is expressed in all stages of male germ cell development during spermatogenesis (spermatogones to ejaculated spermatozoa, Rondena et al., 2005; Figure 5). In contrast, DPL is only detected in spermatids at final stages of spermiogenesis in ovine, human and mice, (Behrens et al., 2002; Espenes et al., 2006; Serres et al., 2006; Figure 5). Interestingly in ram, DPL is observed in spermatids during the elongation process, in Sertoli cells after sperm release and is completely absent in spermatozoa (Espenes et al., 2006). Authors suggest that DPL is present in cytoplasmic lobes of maturing spermatids that culminate in DPL concentration in residual bodies which are subsequently released by spermatids then engulfed by Sertoli cells. DPL seems not to be detected in spermatozoa. However, sperm supplementation with recombinant ovine DPL protein during *in vitro* capacitation, significantly improves spermatozoa motility, vigor, viability and fertilization rate (Pimenta et al., 2012a). At this step we can proposed that DPL expression in ovine ejaculated sperm may be under the threshold of the detection limit of the method and the antibodies used. Otherwise, DPL could be produced by another cell type in the genital tract thus influencing the behavior of spermatozoa. This hypothesis was also suggested in human by Peoc'h and collaborators which completed previous studies by the localization of DPL on the flagella of epididymal and mature spermatozoa, and in seminal plasma (Peoc'h et al., 2002). As DPL seems to be transiently expressed in spermatids but is not detected in differentiated testicular spermatozoa, these authors considered that DPL could be acquired during the maturation of spermatozoa through the epididymis, as it has been described for other GPI-anchored proteins on spermatozoa (Peoc'h et al., 2002). Serres and collaborators reinforced this hypothesis by the observation of a DPL-staining in epithelial cells of the boar epididymis, suggesting a possible epididymal origin of DPL and a potential role during spermatozoa maturation (Serres et al., 2006). The transient presence of DPL in the final stages of spermiogenesis points an important role of this protein in the final remodeling of spermatids prior to their release into the testicular seminiferous lumen. The role of DPL in this spermiogenesis process was completely demonstrated by the analysis of mouse *Prnd* knock-out lines. Indeed, ablation of *Prnd* (*Prnd*^−/−^) in two different mouse lines lead to infertile males with different sperm phenotypes.

The *Prnd*^−/−^ mouse line with a 129/Ola genetic background produced low numbers of spermatozoa with poor motility and abnormal nuclei and acrosomes, greatly affecting the fertilization process. Indeed, sperm from DPL-deficient mice appears to be unable to undergo the normal acrosome reaction that is necessary to penetrate the zona pellucida of the ovum and *Prnd*^−/−^ males are completely sterile (Behrens et al., 2002).

The second *Prnd*^−/−^ mouse line produced on a mixed C57BL6/CBA genetic background produced a normal number of motile spermatozoa but these spermatozoa had an altered chromatin structure and DNA damages that induce an early arrest of embryo development (Paisley et al., 2004). A common phenotype between both *Prnd*^−/−^ mice is a loss of sperm head integrity.

In conclusion, the localization of DPL on both somatic (Sertoli cells) and germinal cells strongly suggests that this protein plays a major role in male fertility. In most species, its expression in testicular germ cells was detected at late stage of spemiogenesis, principally in spermatids with a transient presence in acrosome. These data show that DPL is implicated in normal acrosome genesis and thus in spermatozoa fertilizing ability. Supplemental roles could be attributing to DPL by its presence at earliest stages of testis development in bovine and goats, suggesting an involvement in germinal cell ontogeny. Moreover, DPL has also been detected in goat fetal Leydig cells (steroidogenic cells) where its role remains to be defined (Kocer et al., 2007). Finally some roles of DPL in ovarian differentiation couldn't be discarded since DPL has been detected in female germ cells of goat fetuses and in granulosa cells and follicular fluid in bovine (Rondena et al., 2005; Kocer et al., 2007). This last observation reinforces the idea that DPL may contribute to regulate fertility, since follicular fluid has been shown to influence sperm mobility and fertility (Rodriguez et al., 2001).

In order to pinpoint putative other reproductive roles of DPL, it will be of great interest to engineer *PRND* mutant animals in mammalian species other than mice. For the sex-differentiating process, it is know that the mouse species remains less sensible to gene dosage and haplo-insufficiency than humans. Furthermore, *PRND* expression profiles suggest additional roles for DPL in goat testis differentiation compare to mice (Kocer et al., 2007). Accordingly, *PRND* gene ablation in goats is currently under investigation in our laboratory that has recently succeeded in such technologies (Boulanger et al., 2014).

Three isoforms of human *PRNT* have been described and are exclusively expressed in the adult testis, thus absent in fetal tissues including testis (Makrinou et al., 2002). In goats, *PRNT* is weakly and stochastically expressed in both testes and ovaries at various developmental stages, suggesting that the expression pattern of this gene differs between ruminant and human or, most probably, that ruminant *PRNT* is a pseudogene (Kocer et al., 2007). By contrast, recent results demonstrate that PRT is found in the ram germinal cells. Notably, PRT expression is localized in the nuclei of spermatogonia, spermatocytes, spermatids and in the sperm acrosome. These observations suggest that ovine *PRNT* could be a translated protein-coding gene, pointing to a role for PRT in the ram spermatogenesis, throughout spermatogenic cell proliferation and sperm maturation (Pimenta et al., 2012b). However, it is difficult to conclude on a real role of PRT during spermatogenesis. The lack of PRT detection in others species, supports the hypothesis that PRT could be a pseudogene.

## PRP^c^ and its shadow

In the CNS, PRP^c^, and SHO present a partially reciprocal pattern of expression, suggesting a common function of these two proteins in neuronal cells. This overlapping expression leads to Shadoo protein designation (Shadoo is the Japanese word for shadow) and this protein was considered as the putative host-encoded protein that compensates for the lack of PRP^c^. Although PRP^c^ and SHO are present in gonads, their single and/or common roles are not established in reproductive biology.

**Figure 6:**
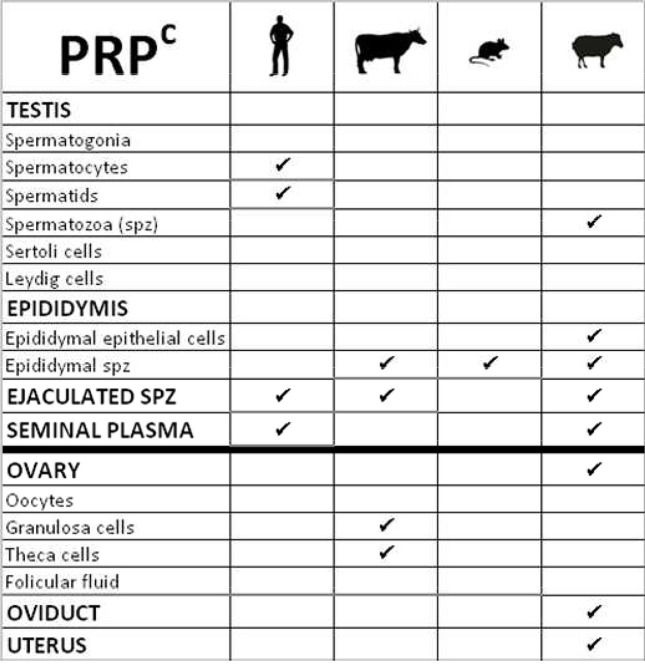
**Table summarizing the cellular localization of Prion protein (PRP^c^) in gonads of various species**.

PRP^c^ has been more studied and was detected on spermatozoa of different species including human, cattle and mouse (Shaked et al., 1999) but the nature of PRP^c^ isoforms on spermatozoa is debated and appears different according to antibodies. A first study showed the presence of PRP^c^ on epididymal sperm extracts from epididymis of mouse and bovine and from ejaculated spermatozoa from bovine and human (Figure 6). The molecular weight of PRP^c^ in epididymal sperm cells was similar to that of the brain (control tissue) whereas in mature sperm cells, the PRP^c^ isoform detected is smaller and C-terminally-truncated. Authors suggested that the C-terminal portion of the PRP^c^ is removed during the process of sperm maturation in epididymis and that the protein is inserted via its N-terminal part in the membrane of ejaculated sperm (Shaked et al., 1999). In contrast, Peoc'h group demonstrated that PRP^c^ was recognized on sperm membranes by antibodies binding to the C-terminus part of the protein, suggesting that only N-terminally truncated fragments of PRP^c^ are present in these cells in the human species. In addition in human testes, 3 isoforms of PRP^c^, an unglycosylated full-length and two N-terminally truncated proteins, are also detected in spermatocytes and spermatids (Peoc'h et al., 2002; Figure 6). Differences in the nature of PRP^c^ truncated (C- or N-term) could be due to differences in the protocol for preparing spermatozoa extracts that could affect proteolysis differentially, or to the antibodies used. Studies in ram supports the work of Shaked and collaborators by demonstrating that one major glycosylated C-terminally truncated PRP^c^ isoform is associated with sperm from testis, cauda epididymis and semen and also in sperm cytoplasmic droplets that are released during maturation (Ecroyd et al., 2004; Figure 6). Other PRP^c^ isoforms were compartmentalized within cauda epididymal fluid and semina plasma (Ecroyd et al., 2004). Indeed, Gatti and colleagues demonstrated the synthesis and secretion of soluble PRP^c^ by the epithelial cells lining the ram epididymis (Figure 6). These cells produce large quantities of a specific isoform of PRP^c^ that seems to be processed post-secretion in different ways during epididymal transit. In ovine spermatozoa, different forms of PRP^c^ have been found, as reported previously for others species (Shaked et al., 1999), that seem to be inserted into the sperm membrane mainly during ejaculation (Gatti et al., 2002). In summary, both glycosylated and proteolytic isoforms of PRP^c^ are present in the male reproductive tract. However, the main isoforms differ between the sperm and the reproductive fluid, suggesting only a low extend of exchange between these two compartments. By studying *Prnp* deficient mice, a protective role of PRP^c^ against copper toxicity has been proposed since sperm cells originating from *Prnp*^−/−^ mice were significantly more susceptible to high copper concentrations than sperm from wild-type mice (Shaked et al., 1999). The presence of an anti-oxidant defense in the sperm-surrounding media is highly important, especially during passage and storage in the epididymis, since these germinal cells lack the molecular machinery to regenerate damaged lipids and proteins. Nevertheless, *Prnp* null mice are fully fertile and the ablation of *Prnp* in *Prnd*^−/−^ mice has no additional effect on the phenotype described for *Prnd*^−/−^ males, suggesting that PRP^c^ is not involved in fertilizing capacity of mice spermatozoa, at least under normal breeding conditions (Büeler et al., 1992; Manson et al., 1994; Paisley et al., 2004).

PRP^c^ is also present in the female reproductive tract. This protein has been detected in the ovary, oviduct and uterus of pregnant and cyclic ewes (Moudjou et al., 2001; Tuo et al., 2001; Figure 6). In bovine, *PRNP* is expressed in both theca and granulosa cells of ovarian follicles notably in developing follicles suggesting that it could promote the growth of dominant follicles (Forde et al., 2008; Figure 6). Again, invalidation of this gene did not induce any noticeable fertility defect in the studied females. However, these animals were kept under control breeding conditions and not challenged, through induction of oxidative stresses for example. Such challenges revealed yet undiscovered function of PrP in other organs, such as placenta (Alfaidy et al., 2013), and such experiments would be of interest to further assess the function of PrP in the gonads.

Although no biological role of SHO has been defined in reproduction, few data are available in mice. Generation of transgenic reporter mice for the gene encoding SHO protein (*Sprn*) has permitted to show a *Sprn*-LacZ expression in the male and female gonads. In both cases, staining was cell-specific, in the interstitial Leydig cells in testis and in granulosa cells of ovarian follicle (Young et al., 2011). Leydig cells are the site of testosterone biosynthesis that is required for the development of the male reproductive system, and the initiation and maintenance of spermatogenesis. Deregulation of some genes expressed in Leydig cells such as proliferin-related protein (PRP) result in decreased testosterone production and has an impact on development of male reproductive system and fertility (Zhao et al., 2011). If we consider the testicular SHO localization, could this protein have the same function in male fertility? Another way to determine the function of a protein is to study the ablation of its coding gene. The lack of *Sprn* in mice has no effect on fertility, as judged by the measurement of the litter size (Daude et al., 2012). Given that DPL is implicated in spermiogenesis and PRP^c^ is present on spermatozoa, one can imagine that these close proteins can compensate each other. *Sprn* invalidation in *Prnd*^−/−^ mice does not increase the testicular phenotype associated with single *Prnd* knockout (our unpublished data). In the same way, mice deficient for both SHO and PRP^c^ were also found to be viable and fertile (Daude et al., 2012). By contrast, the *Sprn* knockdown in *Prnp*^−/−^ mice presents an embryonic lethal phenotype at developmental stage E7.5 (Passet et al., 2012). Discordant results between *Sprn*^KO^/*Prnp*^KO^ and *Sprn*^KD^/*Prnp*^KO^ could be due to different mice genetic backgrounds used in these studies or to a genetic adaptation of the double knockout animals. Nevertheless, gene-targeting experiments in mice do not allow yet defining a putative role of SHO in reproduction.

## Conclusion

Among the prion-gene family members, *PRND* is yet the sole to have been clearly linked to reproductive biology with a crucial function during the late steps of spermatogenesis. Indeed its gene ablation in mice leads to male infertility. Although expression of the other three members *PRNP*. *PRNT*, and *SPRN* has been demonstrated at mRNA and protein levels in gonads and/or reproductive tracts, their specific reproductive functions, if any, remain to be elucidated. The fact that these putative functions haven't been pointed on by human genetics or gene-targeting in other mammalian species may suggest that these proteins by alone have no critical reproductive functions or that their functions aren't hugely affected by mutational events. Indeed, even if the physiological role of PRP^c^ remains unclear in the central nervous system, according to the fact that *Prnp*^−/−^ mice or goats seem to be unaffected and well-being, it is a mis-folding of PRP^c^ (PRP^sc^) that remains highly detrimental for the central nervous system physiology (a sort of gain-of-function mutation). This means that we cannot exclude some detrimental reproductive effects of these proteins without any mutational changes, but with post-translational and/or conformational changes remaining very difficult to pin-point.

### Conflict of interest statement

The authors declare that the research was conducted in the absence of any commercial or financial relationships that could be construed as a potential conflict of interest.

## References

[B1] Alfaidy N. Chauvet S. Donadio-Andrei S. Salomon A. Saoudi Y. Richaud P.. (2013). Prion protein expression and functional importance in developmental angiogenesis: role in oxidative stress and copper homeostasis. Antioxid. Redox Signal. 18, 400–411. 10.1089/ars.2012.4637 22861352

[B2] Behrens A. Genoud N. Naumann H. Rülicke T. Janett F. Heppner F. L.. (2002). Absence of the prion protein homologue Doppel causes male sterility. EMBO J. 21, 3652–3658. 10.1093/emboj/cdf386 12110578 PMC125402

[B3] Bendheim P. E. Brown H. R. Rudelli R. D. Scala L. J. Goller N. L. Wen G. Y.. (1992). Nearly ubiquitous tissue distribution of the scrapie agent precursor protein. Neurology 42, 149–156. 10.1212/WNL.42.1.149 1346470

[B4] Boulanger L. Pannetier M. Gall L. Allais-Bonnet A. El Zaiat M. Le Bourhis D.. (2014). FOXL2 is a female sex-determining gene in the goat. Curr. Biol. 24, 404–408. 10.1016/j.cub.2013.12.039 24485832

[B5] Büeler H. Fischer M. Lang Y. Bluethmann H. Lipp H. P. DeArmond S. J.. (1992). Normal development and behaviour of mice lacking the neuronal cell-surface PrP protein. Nature 356, 577–582. 10.1038/356577a0 1373228

[B6] Comincini S. Foti M. G. Tranulis M. A. Hills D. Di Guardo G. Vaccari G.. (2001). Genomic organization, comparative analysis, and genetic polymorphisms of the bovine and ovine prion Doppel genes (*PRND*). Mamm. Genome 12, 729–733. 10.1007/s00335-001-2064-4 11641722

[B7] Daude N. Westaway D. (2011). Biological properties of the PRP-like Shadoo protein. Front. Biosci. 16, 1505–1516. 10.2741/3801 21196244

[B8] Daude N. Wohlgemuth S. Brown R. Pitstick R. Gapeshina H. Yang J.. (2012). Knockout of the prion protein (PrP)-like *Sprn* gene does not produce embryonic lethality in combinaison with PrP^c^-deficiency. Proc. Natl. Acad. Sci. U.S.A. 109, 9035–9040. 10.1073/pnas.1202130109 22619325 PMC3384183

[B9] DeFalco T. Capel B. (2009). Gonad morphogenesis in vertebrates: divergent means to a convergent end. Annu. Rev. Cell Dev. Biol. 25, 457–482. 10.1146/annurev.cellbio.042308.13350 19807280 PMC4507502

[B11] Ecroyd H. Sarradin P. Dacheux J. L. Gatti J. L. (2004). Compartmentalization of prion isoforms within the reproductive tract of the ram. Bio. Reprod. 71, 993–1001. 10.1095/biolreprod.104.029801 15163617

[B12] Espenes A. Harbitz I. Skogtvedt S. Fuglestveit R. Berg K. A. Dick G.. (2006). Dynamic expression of the prion-like protein Doppel in ovine testicular tissue. Int. J. Androl. 29, 400–408. 10.1111/j.1365-2605.2005.00618.x 16390495

[B13] Essalmani R. Taourit S. Besnard N. Vilotte J. L. (2002). Sequence determination and expression of the ovine doppel-encoding gene in transgenic mice. Gene 285, 287–290. 10.1016/S0378-1119(02)00391-8 12039056

[B14] Forde N. Rogers M. Canty M. J. Lonergan P. Smith G. W. Coussens P. M.. (2008). Association of the prion protein and its expression with ovarian follicle development in cattle. Mol. Reprod. Dev. 75, 243–249. 10.1002/mrd.20807 17595008

[B15] Gatti J. L. Métayer S. Moudjou M. Andréoletti O. Lantier F. Dacheux J. L.. (2002). Prion protein is secreted in soluble forms in the epididymal fluid and proteolytically processed and transported in seminal plasma. Biol. Reprod. 67, 393–400. 10.1095/biolreprod67.2.393 12135872

[B16] Harris D. A. (1999). Cell biological studies of the prion protein. Curr. Issues Mol. Biol. 1, 65–75. 11475702

[B17] Harrison P. M. Khachane A. Kumar M. (2010). Genomic assessment of the evolution of the prion protein gene family in vertebrates. Genomics 95, 268–277. 10.1016/j.ygeno.2010.02.008 20206252

[B18] Kashimada K. Koopman P. (2010). Sry: the master switch in mammalian sex determination. Development 137:3921–3930 10.1242/dev.048983 21062860

[B19] Kocer A. Gallozzi M. Renault L. Tilly G. Pinheiro L. Le Provost F.. (2007). Goat *PRND* expression pattern suggests its involvement in early sex differentiation. Dev. Dyn. 236, 836–842. 10.1002/dvdy.21066 17226816

[B20] Lee I. Y. Westaway D. Smit A. F. Wang K. Seto J. Chen L.. (1998). Complete genomic sequence and analysis of the prion protein gene region from three mammalian species. Genome Res. 8, 1022–1037. 9799790 10.1101/gr.8.10.1022

[B21] Li A. Sakaguchi S. Shigematsu K. Atarashi R. Roy B.C. Nakaoke R.. (2000). Physiological expression of the gene for PrP-like protein, PrPLP/Dpl, by brain endothelial cells and its ectopic expression in neurons of PrP-deficient mice ataxic due to Purkinje cell degeneration. Am. J. Pathol. 157, 1447–1452. 10.1016/S0002-9440(10)64782-7 11073804 PMC1885740

[B22] Makrinou E. Collinge J. Antoniou M. (2002). Genomic characterization of the human protein (PrP) gene locus. Mamm. Genome 13, 696–703. 10.1007/s00335-002-3043-0 12514748

[B23] Manson J. C. Clarke A. R. Hopper M. L. Aitchison L. McConnell I. Hope J. (1994). 129/Ola mice carrying a null mutation in PrP that abolishes mRNA production are developmentally normal. Mol. Neurobiol. 8, 121–127. 10.1007/BF02780662 7999308

[B24] Moore R. C. Lee I. Y. Silverman G. L. Harrison P. M. Strome R. Heinrich C.. (1999). Ataxia in prion protein (PrP)-deficient mice is associated with upregulation of the novel PrP-like protein doppel. J. Mol. Biol. 292, 797–817. 10.1006/jmbi.1999.3108 10525406

[B25] Moudjou M. Frobert Y. Grassi J. La Bonnardière C. (2001). Cellular prion protein status in sheep: tissue-specific biochemical signatures. J. Gen. Virol. 82, 2017–2024. 10.1099/vir.0.17776-0 11458009

[B26] Paisley D. Banks S. Selfridge J. McLennan N. F. Ritchie A. M. McEwan C.. (2004). Male infertility and DNA damage in Doppel knockout and prion protein/Doppel double-knoukout mice. Am. J. Pathol. 164, 2279–2288. 10.1016/S0002-9440(10)63784-4 15161660 PMC1615753

[B27] Passet B. Young R. Makhzami S. Vilotte M. Jaffrezic F. Halliez S.. (2012). Prion protein and Shadoo are involved in overlapping embryonic pathways and trophoblastic development. PLoS ONE 7:e41959. 10.1371/journal.pone.0041959 22860039 PMC3408428

[B28] Peoc'h K. Serres C. Frobert Y. Martin C. Lehmann S. Chasseigneaux S.. (2002). The human “prion-like” protein Doppel is expressed in both Sertoli cells ans spermatozoa. J. Biol. Chem. 277, 43071–43078. 10.1074/jbc.M206357200 12200435

[B29] Pimenta J. Dias F. M. Marques C. C. Baptista M. C. Vasques M. I. Horta A. E.. (2012a). The prion-like protein Doppel enhances ovine spermatozoa fertilizing ability. Reprod. Domest. Anim. 47, 196–202. 10.1111/j.1439-0531.2011.01827.x 21806689

[B10] Pimenta J. Domingos A. Santos P. Marques C. C. Cantante C. Santos A.. (2012b). Is prnt a pseudogene? Identification of ram Prt in testis and ejaculated spermatozoa. PLoS ONE 7:e42957. 10.1371/journal.pone.0042957 22937002 PMC3427297

[B30] Premzl M. Gamulin V. (2007). Comparative genomic analysis of prion genes. BMC Genomics 8:1. 10.1186/1471-2164-8-1 17199895 PMC1781936

[B31] Premzl M. Gready J. E. Jermiin L. S. Simonic T. Marshall Graves J. A. (2004). Evolution of vertebrate genes related to prion and shadoo proteins—clues from comparative genomic analysis. Mol. Bio. Evol. 21, 2210–2231. 10.1093/molbev/msh245 15342797

[B32] Premzl M. Sangiorgio L. Strumbo B. Marshall Graves J. A. Simonic T. Gready J. E. (2003). Shadoo, a new protein highly conserved from fish to mammals and with similarity to prion protein. Gene 314, 89–102. 10.1016/S0378-1119(03)00707-8 14527721

[B33] Prusiner S. B. (1998). Prions. Proc. Natl. Acad. Sci. U.S.A. 95, 13363–13383. 10.1073/pnas.95.23.13363 9811807 PMC33918

[B34] Rodriguez H. Torres C. Valdes X. Guerra H. Pastor L. M. Maccallini G.. (2001). The acrosomic reaction in stallion spermatozoa: inductive effect of the mare preovulatory follicular fluid. Biocell. 25, 115–120. 11590887

[B35] Rondena M. Ceciliani F. Comazzi S. Pocacqua V. Bazzocchi C. Luvoni C.. (2005). Identification of bovine doppel protein in testis, ovary and ejaculated spermatozoa. Theriogenology 63, 1195–1206. 10.1016/j.theriogenology.2004.06.009 15710203

[B36] Saeki K. Matsumoto Y. Hirota Y. Matsumoto Y. Onodera T. (1996). Three-exon structure of the gene encoding the rat prion protein and its expression in tissues. Virus Genes 12, 15–20. 10.1007/BF00369996 8879116

[B37] Schmitt-Ulms G. Ehsani S. Watts J. C. Westaway D. Wille H. (2009). Evolutionary descent of prion genes from the ZIP family of metal ion transporters. PLoS ONE 4:e7208. 10.1371/journal.pone.0007208 19784368 PMC2745754

[B38] Serres C. Peoc'h K. Courtot A. M. Lesaffre C. Jouannet P. Laplanche J. L. (2006). Spatio-developmental distribution of the prion-like protein doppel in Mammalian testis: a comparative analysis focusing on its presence in the acrosome of spermatids. Biol. Reprod. 74, 816–823. 10.1095/biolreprod.105.047829 16421231

[B39] Shaked Y. Rosenmann H. Talmor G. Gabizon R. (1999). A C-terminal-truncated PrP isoform is present in mature sperm. J. Biol. Chem. 274, 32153–32158. 10.1074/jbc.274.45.32153 10542251

[B40] Sprando R. L. Russell L. D. (1987). Comparative study of cytoplasmic elimination in spermatids of selected mammalian species. Am. J. Anat. 178, 72–80. 10.1002/aja.1001780109 3825964

[B41] Tanji K. Saeki K. Matsumoto Y. Takeda M. Hirasawa K. Doi K.. (1995). Analysis of PRPc mRNA by *in situ* hybridization in brain, placenta, uterus and testis of rats. Intervirology 38, 309–315. 8880380 10.1159/000150457

[B42] Tranulis M. A. Espenes A. Comincini S. Skretting G. Harbitz I. (2001). The PrP-like protein Doppel gene in sheep and cattle: cDNA sequence and expression. Mamm. Genome 12, 376–379. 10.1007/s003350010285 11331946

[B43] Tuo W. Zhuang D. Knowles D. P. Cheevers W. P. Sy M. S. O'Rourke K. I. (2001). Prp-c and Prp-sc at the fetal maternal interface. J. Biol. Chem. 276, 18229–18234. 10.1074/jbc.M008887200 11274195

[B44] Watts J. C. Westaway D. (2007). The prion protein family: diversity, rivalry, and dysfunction. Biochim. Biophys. Acta 1772, 654–672. 10.1016/j.bbadis.2007.05.001 17562432

[B45] Weber J. E. Russell L. D. (1987). A study of intercellular bridges during spermatogenesis in the rat. Am. J. Anat. 180, 1–24. 10.1002/aja.1001800102 3661461

[B46] Westaway D. (2004). Inherited prion diseases, in Prion Biology and Diseases, ed Prusiner S. B. (New York, NY: Cold Spring Harbor Laboratory Press), 673–775

[B47] Yoshimoto J. Iinuma T. Ishiguro N. Horiuchi M. Imamura M. Shinagawa M. (1992). Comparative sequence analysis and expression of bovine PrP gene in mouse L-929 cells. Virus Genes 6, 343–356. 10.1007/BF01703083 1362024

[B48] Young R. Le Guillou S. Tilly G. Passet B. Castille J. Beringue V.. (2011). Generation of *Sprn*-regulated reporter mice reveals gonadic spatial expression of the prion-like protein Shadoo in mice. Biochem. Biophys. Res. Commun. 412, 752–756. 10.1016/j.bbrc.2011.08.049 21871438

[B49] Zhao L. Hao J. Hu J. Wang Q. Lü Z. Wang L.. (2011). Expression of proliferin-related protein in testis and the biological significance in testosterone production. Mol. Cell. Endocrinol. 343, 25–31 10.1016/j.mce.2011.05.046 21679748

